# Supplementary material for: Imiquimod, a Promising Broad-Spectrum Antiviral, Prevents SARS-CoV-2 and Canine Coronavirus Multiplication Through the MAPK/ERK Signaling Pathway
Source: Viruses. 2025 May 31;17(6):801. doi: 10.3390/v17060801 (PMC12197677; doi:10.3390/v17060801)
Supplement: Supplementary file 1 [file viruses-17-00801-s001.zip › viruses-3633604-supplementary.pdf]

Supplementary Materials:

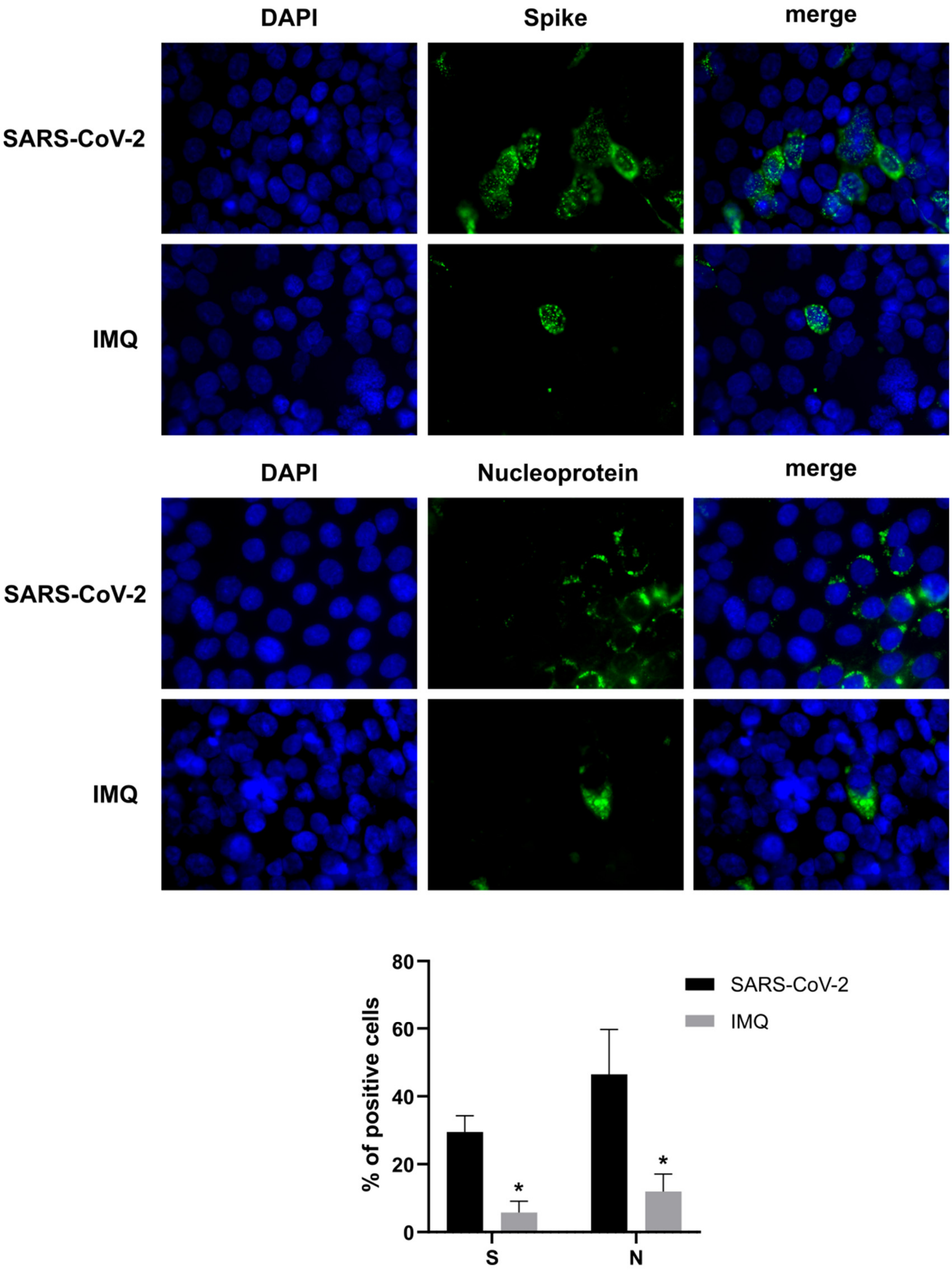

Supplementary Figure S1. Effect of IMQ on SARS-CoV-2 protein expression. Calu-3 cells were infected with SARS-CoV-2 (moi = 0.1) and treated or not with IMQ (10 µl/ml) for 24 h. Viral protein expression was assessed by IF staining to detect the intracellular localization of Spike glycoprotein (S) and Nucleocapsid protein (N) of SARS-CoV-2 (labeled in green); nuclei were stained with DAPI (blue). Magnification = 400X. *Significantly different from SARS-CoV-2 (p-value<0.05); unpaired t-test.

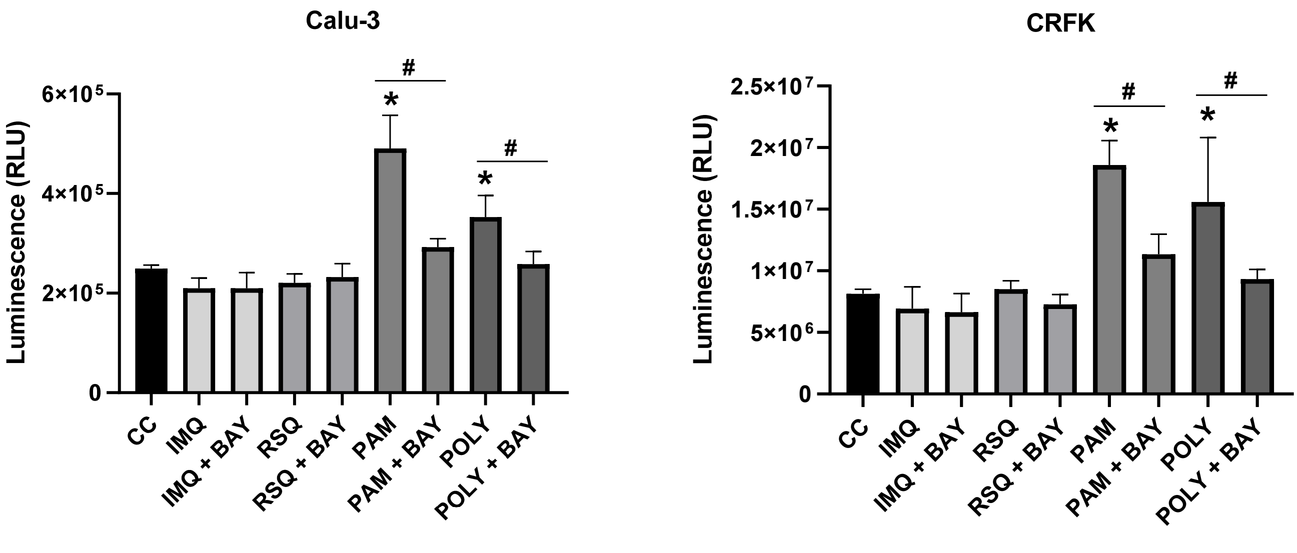

Supplementary Figure S2. Effect of TLR agonists on transcriptional activation of NF-κB. Calu-3 and CRFK cells were transfected with NF-κB-luciferase reporter plasmid and β-galactosidase control plasmid. After 24 h, cells were stimulated or not with IMQ (10 μg/ml), RSQ (10 μg/ml), Pam2CSK4 (PAM, 100 ng/ml), poly(I:C)-HMW (POLY, 10 μg/ml) and BAY 11-7082 (10 μg/ml) for 6h. Luciferase activity was measured in cell extracts, and each value was normalized to β-galactosidase activity in relative luciferase units (RLUs). (CC): unstimulated control cells. Data represent mean ± SD for n = 3 independent experiments, performed in duplicate. *Significantly different from CC (p-value<0.05), #Significantly different from TLR agonist (p-value<0.05); One way ANOVA with Dunnett's post test.

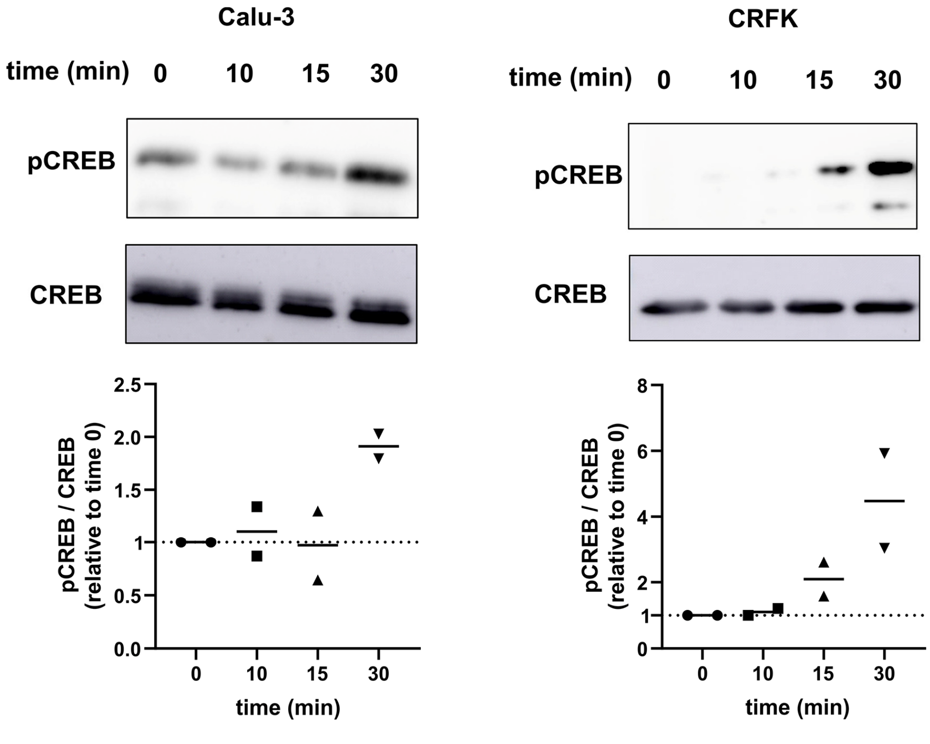

Supplementary Figure S3. Effect of IMQ on CREB phosphorylation. Immunoblot images of p-CREB and CREB expression, and quantitative densitometric analysis in Calu-3 and CRFK cells after exposure or not to IMQ (10 μg/ml) during the indicated times. Data represent mean ± SD for n = 2 independent experiments.
